# *Tn5* transposition in *Escherichia coli* is repressed by Hfq and activated by over-expression of the small non-coding RNA SgrS

**DOI:** 10.1186/s13100-014-0027-z

**Published:** 2014-11-30

**Authors:** Joseph A Ross, Ryan S Trussler, Morgan D Black, Crystal R McLellan, David B Haniford

**Affiliations:** Department of Biochemistry, University of Western Ontario, London, ONN6A 5C1 Canada

**Keywords:** *Tn5*/*IS50*, Hfq, Crp, SgrS, DNA transposition

## Abstract

**Background:**

Hfq functions in post-transcriptional gene regulation in a wide range of bacteria, usually by promoting base pairing of mRNAs with *trans*-encoded sRNAs. It was previously shown that Hfq down-regulates *Tn10* transposition by inhibiting *IS10* transposase expression at the post-transcriptional level. This provided the first example of Hfq playing a role in DNA transposition and led us to ask if a related transposon, *Tn5*, is similarly regulated.

**Results:**

We show that Hfq strongly suppresses *Tn5* transposition in *Escherichia coli* by inhibiting *IS50* transposase expression. However, in contrast to the situation for *Tn10*, Hfq primarily inhibits *IS50* transposase transcription. As Hfq does not typically function directly in transcription, we searched for a transcription factor that also down-regulated *IS50* transposase transcription and is itself under Hfq control. We show that Crp (cyclic AMP receptor protein) fits these criteria as: (1) disruption of the *crp* gene led to an increase in *IS50* transposase expression and the magnitude of this increase was comparable to that observed for an *hfq* disruption; and (2) Crp expression decreased in *hfq*^*−*^. We also demonstrate that *IS50* transposase expression and *Tn5* transposition are induced by over-expression of the sRNA SgrS and link this response to glucose limitation.

**Conclusions:**

*Tn5* transposition is negatively regulated by Hfq primarily through inhibition of *IS50* transposase transcription. Preliminary results support the possibility that this regulation is mediated through Crp. We also provide evidence that glucose limitation activates *IS50* transposase transcription and transposition.

**Electronic supplementary material:**

The online version of this article (doi:10.1186/s13100-014-0027-z) contains supplementary material, which is available to authorized users.

## Background

Transposase proteins catalyze the chemical steps in bacterial transposition reactions. It follows that the regulation of expression of these genes is a critical feature in dictating the transposition frequency of most transposons. In many instances, including *Tn10*/*IS10* and *Tn5*/*IS50*, transposase gene promoters are inherently weak. In addition, DNA adenine methylase (DAM) limits initiation of *IS10* and *IS50* transposase gene transcription by methylating promoter elements [1,2]. These factors together make transcription initiation a limiting step in *Tn10*/*IS10* and *Tn5*/*IS50* transposition reactions [3,4]. There are also examples where translation of transposase transcripts is subject to both intrinsic and host levels of regulation. In the case of *IS10* transposase, the ribosome binding site is inherently weak and the transposon encodes an antisense RNA that binds the translation initiation region (TIR), blocking ribosome binding [5,6]. There is also evidence that the ‘host’ protein Hfq helps mediate the pairing interaction between the antisense RNA and the *IS10* transposase transcript [7,8].

**Figure 1:**
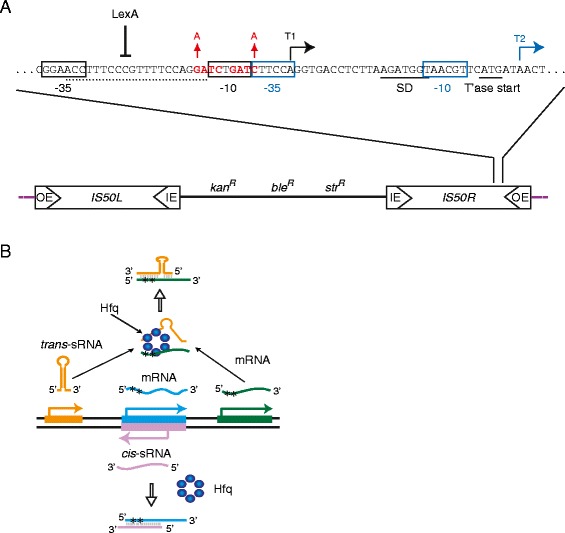
***Tn5*** **/** ***IS50*** **structure and gene expression. (A)** The structure of *Tn5* is shown along with transcription units within *IS50*-Right. There are two distinct promoters defined by -35/-10 regions that control transposase (black) and inhibitor (blue) expression. T1 is the transposase transcript and T2 is the inhibitor transcript. The Shine-Dalgarno sequence of T1 is also shown. Expression of T1 but not T2 is regulated by DAM methylation at two GATC sequences (red) and potentially LexA binding (dotted line defines a putative LexA binding site). Mutations in the *dam* sites used in this work are shown. *kan* ^*R*^, *ble* ^*R*^ and *str* ^*R*^ are kanamycin, bleomycin and streptomycin resistance genes, respectively. **(B)** Post-transcriptional regulation by Hfq. Hfq (blue hexamer) is shown catalyzing the pairing of an sRNA with an mRNA. The sRNA can be either *cis* or *trans* encoded relative to its target mRNA. In both cases the sRNA is shown pairing to the translation initiation region of the mRNA (asterisks) and would block translation.

Hfq is a global regulator of gene expression in bacteria. It typically functions at the post-transcriptional level, influencing translation initiation and/or transcript stability by catalyzing the pairing of small RNAs (sRNA) and their mRNA targets (Figure 1B and reviewed in [9]). In contrast to the many examples of Hfq acting in a post-transcriptional capacity to impact gene expression, there is (to our knowledge) only one example in the literature of Hfq acting at the level of transcription to influence gene expression. In the case of ribosomal proteins rpsO, rpsT and rpsB-tsf, Hfq was shown to increase transcript levels without influencing transcript stability. It was suggested that this is accomplished through Hfq binding to secondary structure elements in the respective transcripts that form early in the elongation phase of transcription and that this interaction reduces RNA polymerase pausing [10].

As noted above, Hfq has been implicated in the regulation of *Tn10*/*IS10* transposition. Under conditions of *hfq* deficiency, a large increase in both *Tn10*/*IS10* transposition (up to 80-fold) and transposase expression (up to 7-fold) were observed. The existing evidence is consistent with Hfq acting as a negative regulator of *IS10* transposase expression by both antisense dependent and independent pathways. In support of the latter, it was found that *hfq* deficiency (or *hfq*^*−*^) had a significant impact on *Tn10* transposition even when the level of antisense RNA was insufficient to impact on transposase expression (that is when *Tn10* is present in single copy in the bacterial chromosome). In addition, there was a synergistic increase in transposase expression when both *hfq* and the antisense RNA were knocked out, implying that Hfq does not function exclusively in the same pathway as the antisense RNA [7].

Taking the above results into account, and considering that most bacterial transposition systems are not regulated by antisense RNAs, we wondered if Hfq might play a more general role in regulating transposition systems. In the current work, we tested this hypothesis by asking if *Tn5* transposition is also regulated by Hfq. Like *Tn10*, *Tn5* is a composite transposon (Figure 1A). The two transposons are closely related but *Tn5* lacks an antisense RNA regulatory system and consequently if Hfq were to regulate this system at the post-transcriptional level, it is likely that a trans-encoded sRNA would play a role [11-13]. Tn5 does encode an inhibitor protein that limits *Tn5*/*IS50* transposition by dimerizing with the transposase protein, forming an inactive complex [14]. Transposase and the inhibitor protein are expressed from overlapping promoters, P1 and P2 (color coded in Figure 1A), with the inhibitor transcript (T2) being expressed at a higher level than the transposase transcript (T1). T1 expression is down-regulated by DAM (reviewed in [15]). There is some evidence that P1 is also negatively regulated by LexA, an SOS-inducible transcriptional repressor [16]. However, there is little else known with regard to host proteins that influence either transposase transcription or translation.

In the current work, we show that both *Tn5* transposition and *IS50* transposase expression increase significantly in *E. coli* under conditions of *hfq* deficiency. However, unlike the situation in *Tn10*/*IS10* transposition, the up-regulation of *IS50* transposase expression appears mainly to be due to an increase in transposase gene transcription. As Hfq does not typically function directly in transcription, we looked at the possibility that Hfq regulates *IS50* transposase expression by controlling the expression of a transcription factor. Towards this end, we provide evidence that Hfq acts in a regulatory network with Crp (cyclic AMP receptor protein) to down-regulate *IS50* transposase transcription. Finally, we demonstrate that over-expression of an sRNA (SgrS) activates expression of the *IS50* transposase gene specifically when cells are grown with glucose as the sole carbon source. Evidence is presented that this up-regulation is a consequence of glucose limitation, demonstrating that the *IS50* transposase promoter (and *Tn5* transposition) is responsive to the nutrient status of the cell.

## Results

### Hfq is a potent negative regulator of *Tn5* transposition

**Figure 2:**
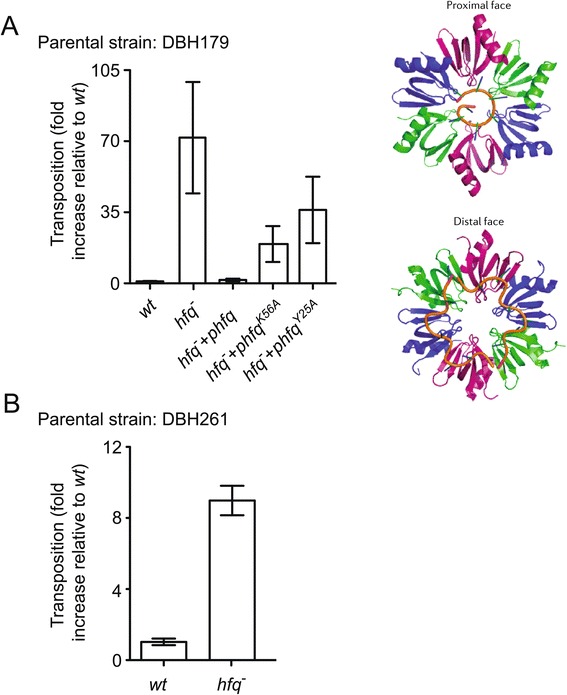
**Frequencies of** ***Tn5*** **transposition in** ***hfq*** ^**−**^ **versus** ***wt*** **strains of** ***E. coli*** **. (A)** *Tn5* transposition from the chromosome of DBH179 and derivatives (*hfq* ^−^ and *dam* ^−^) was measured by the conjugal ‘mating out’ assay as described in Methods. For purposes of *trans*-complementation, strains contained an empty vector or a low-copy plasmid encoding either wild type *hfq* ^*WT*^ or mutant forms of *hfq* (K56A or Y25A) expressed from the *hfq P3* promoter. The data was compiled from four independent experiments, each with at least three isolates of each strain. The average transposition frequency was 8.33 × 10^−5^ events per mL of mating mix for the *wt* strain (no ‘*hfq* plasmid’) and for purposes of comparison this value was set at 1 and all other values normalized to this. The illustration shows the structure of an Hfq hexamer with RNA (gold) bound either to the proximal or distal face [9]. The Y25A mutation inhibits RNA binding to the distal face and the K56A mutation inhibits RNA binding to the proximal face. Adapted from Nature Reviews: Microbiology [9] with permission from Macmillan Publishers. **(B)** *Tn5* transposition from the chromosome of DBH261 and derivatives (*hfq* ^−^ and *dam* ^−^) was measured as in **(A)**. The data shown is from one experiment with five independent isolates of each strain. The average transposition frequency for the *wt* strain was 2.57 × 10^−6^ events per mL of mating mix. In **(A)** and **(B)** the error bars indicate standard error of the mean.

We asked if Hfq regulates *Tn5* transposition in *E. coli* by measuring the frequency of *Tn5* transposition under conditions of *hfq* deficiency using the ‘mating out’ assay. In this assay, an F^+^ donor strain harboring a chromosomal copy of *Tn5* was mated to an F^−^ recipient strain and the mating efficiency and number of transposition events were measured by plating mating mixes on the appropriate selective media (see Methods). We show in Figure 2A that in one donor strain background (DBH179) *Tn5* transposition increased by close to 75-fold under conditions of *hfq* deficiency. Note that we did not have a defective copy of *Tn5* to act as a negative control in this experiment. *In lieu* of this, we carried out physical mapping on a sampling of colonies present on ‘hop’ plates to ensure that *bona fide* transposition events were being measured in both *wt* and *hfq*^−^ strains (Additional file 1).

We also performed a complementation assay in the DBH179 strain background to further test that the increase in transposition reported above in *hfq*^−^ was actually due to the absence of Hfq, as opposed to possible polar effects of the *hfq* disruption allele. Towards this end, we introduced *hfq* on a low-copy plasmid (pDH700) into the *hfq*^−^ strain and measured *Tn5* transposition as above. We observed nearly complete complementation by plasmid-borne *hfq,* as transposition was reduced approximately 45-fold relative to when no *hfq* was present (Figure 2A). Furthermore, plasmid-encoded variants of *hfq*, including K56A and Y25A, which are impaired for RNA-binding at the ‘proximal’ and ‘distal’ surface, respectively, failed to complement *hfq* deficiency [17]. This confirms that specific functions of Hfq, namely interaction with RNA via known RNA-binding surfaces, are required for effective repression of *Tn5* transposition.

We also tested the impact of *hfq* deficiency on *Tn5* transposition in a second donor strain background (DBH261) via the ‘mating out’ assay (Figure 2B). In this experiment *hfq*^−^ also caused an increase in *Tn5* transposition, although the magnitude of the effect was smaller (approximately 9-fold) than reported for the DBH179 strain background.

### *IS50* transposase expression increases in *hfq*^*−*^ cells

**Figure 3:**
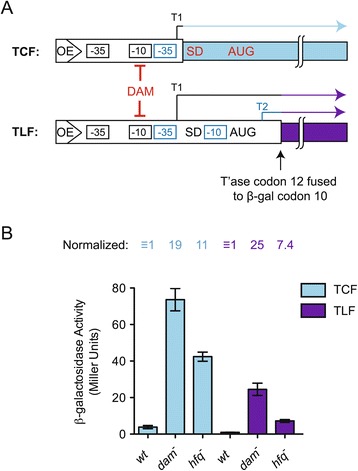
**Transposase-** ***lacZ*** **translational and transcriptional fusion reporter assays in** ***wt*** **,** ***dam*** ^**−**^ **and** ***hfq*** ^**−**^ **strains. (A)** Schematic of the *IS50*-*lacZ* transcriptional fusion (TCF; upper) and translational fusion (TLF; lower) reporters. The TCF reporter encodes the first 80 bp of *IS50-*Right (white rectangle) fused to *lacZ* (light blue rectangle). This fusion encodes only the first 15 nucleotides of the transposase (T1) transcript, which is expressed from the native promoter; the -35/-10 elements are shown in black. The inhibitor transcript is not expressed as the promoter for the inhibitor is missing its -10 region. The TLF encodes the first 128 bp of *IS50-*Right. This includes up to the 12^th^ codon of T1, which is fused in-frame to the 10^th^ codon of *lacZ* (purple rectangle). T1 and T2 and their respective promoter elements (-35/-10 sequences) are color-coded. Note that the start codon for the inhibitor protein has been mutated so that only transposase expression will give rise to β-galactosidase activity. Also note that the transposase promoter in both the TCF and the TLF is sensitive to Dam methylation. **(B)** β-galactosidase activity (given in Miller Units) for isogenic strains (*wt*, *dam* ^−^ or *hfq* ^−^) harboring either the TCF or TLF in single-copy in the chromosome of *E. coli*. For each fusion, the activity was normalized to that of the *wt* strain. The data sets shown for the TCF and TLF were compiled from two and three independent experiments, respectively, with each experiment including at least three replicates. Mean and standard error values are shown.

We next asked if *hfq* status influenced *IS50* transposase expression. In one approach, we measured transposase expression by constructing *IS50*-*lacZ* transcriptional and translational fusions (‘TCF’ and ‘TLF’, respectively; see Figure 3A for schematics), integrating these reporters into the chromosome of a *lac*^−^*E. coli* strain (DBH107), and then performing β-galactosidase assays. This was done for each reporter in isogenic strains that were either *wt*, *dam*^−^ or *hfq*^−^. As expected for a promoter that is DAM-sensitive, transposase expression increased in the context of both transcriptional and translational fusions in the *dam*^−^ strain relative to *wt* (approximately19- and 25-fold, respectively; Figure 3B). The increase in transposase expression for both constructs in *dam*^−^ is indicative of expression coming predominantly from the *P1* promoter [2]. Transposase expression in TCF and TLF constructs also increased in *hfq*^−^ cells (11-fold and 7.4-fold, respectively), indicating that Hfq (or a factor under Hfq control) represses *IS50* transposase expression. As the TCF encodes only 15 nucleotides of the transposase transcript (T1), it seemed most likely that up-regulation of transposase expression in *hfq*^−^ was primarily due to enhanced transcription in both TCF and TLF constructs.

**Figure 4:**
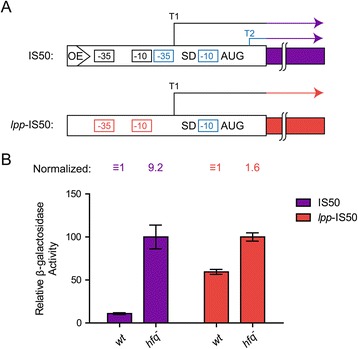
**Heterologous promoter-transposase-** ***lacZ*** **translational fusion reporter assays in** ***wt*** **and** ***hfq*** ^**−**^ **strains. (A)** Schematic of the *IS50*-*lacZ* translational fusion with *IS50* transposase and *lpp* promoters. The *IS50* translational fusion (TLF) is as described in Figure 3A. The *lpp*-*IS50* TLF contains the *lpp* promoter (-35 and -10 elements) fused to the *IS50* transposase gene such that only *IS50* sequences at, and downstream of the T1 transcriptional start, are present. **(B)** β-galactosidase activity for isogenic strains (*wt* or *hfq* ^−^) harboring the indicated TLF on a multicopy plasmid. For each fusion, the activity was normalized to that of the *wt* strain. The data sets shown were compiled from two independent experiments, respectively, with each experiment including at least three replicates. Mean and standard error values are shown.

To further test this possibility, we constructed a TLF reporter (pDH908) wherein the *IS50* transposase promoter was replaced by a heterologous promoter (from the *lpp* gene) whose regulation is not sensitive to *hfq* status [10]. An isogenic plasmid (pDH795) in which the TLF contained the *IS50* transposase promoter was also constructed. Cells (*wt* or *hfq*^*−*^) were transformed with plasmids containing these constructs and reporter expression was measured as above. The results presented in Figure 4 show that transposase expression increased approximately 9-fold for the construct containing the *IS50* promoter and less than 2-fold for the construct containing the *lpp* promoter. These results support our contention that Hfq-directed regulation of *IS50* transposase expression occurs at the transcriptional level because the absence of the *IS50* promoter and not the presence of the *IS50* 5′ UTR was the dominant factor in observing strong up-regulation of reporter expression under conditions of *hfq* deficiency.

### Hfq impacts steady-state levels of full-length *IS50* transposase mRNA

**Figure 5:**
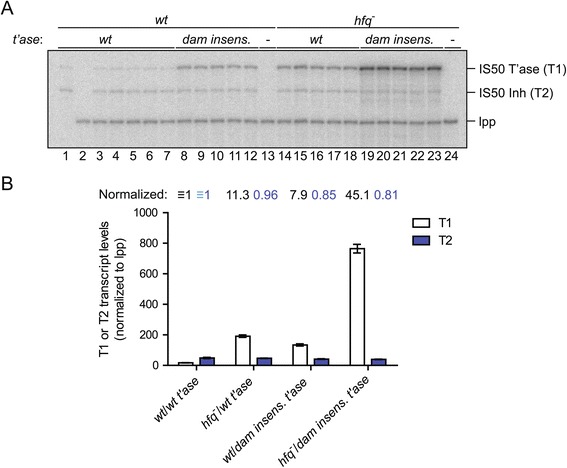
**Steady-state levels of** ***IS50*** **transposase mRNA in** ***wt*** **and** ***hfq*** ^***−***^ **cells. (A)** Plasmids encoding *wt* or DAM-Insensitive *IS50* transposase genes were transformed into *wt* (DBH33) or *hfq* ^*−*^ (DBH16) *E. coli* strains. Total RNA was isolated from five different clones grown to mid-log phase for each of the indicated strains. Primer extension reactions were multiplexed using ^32^P-labeled primers complimentary to *IS50* transposase (primer oDH230) and *lpp* (primer oDH390) RNAs. The corresponding cDNAs were analyzed on a 10% sequencing gel. T1 and T2 are defined in Figure 1. Note that transcription of *lpp* is known to be insensitive to *hfq* status [10]. **(B)** Summary of data in **(A)**.

To further assess the impact of *hfq* deficiency on transposase gene expression, we looked at both the steady-state level and the stability of the transposase transcript (T1) in *hfq*^*+*^ and *hfq*^*−*^ cells. For the steady-state analysis, total RNA was isolated from various strains (*wt* or *hfq*^*−*^) (DBH33 background) containing a multi-copy plasmid encoding the full-length transposase gene under the control of its native promoter. In addition to the *wt* version of this plasmid (pDH533), we also analyzed a mutant form containing mutations in the overlapping *dam* methylation sites in the transposase promoter (pDH752) (see Figure 1A); these mutations make this construct DAM insensitive. Primer extension was used to detect both T1 and T2 transcripts, as well as the lpp transcript (loading control). As expected for a *dam*-sensitive promoter, levels of T1 increased substantially (approximately 8-fold) in *wt* cells containing the plasmid with the *dam*-insensitive promoter versus *wt* cells containing the *wt* promoter (compare lanes 3 to 7 with lanes 8 to 12 in Figure 5A and bar graph in Figure 5B). In contrast, there was no significant change in T2 levels in the above samples. In *hfq*^*−*^ (*wt* promoter) there was also a substantial increase in T1 levels (11-fold) versus the *wt* strain (compare lanes 3 to 7 with lanes 14 to 18) and no significant change in T2 levels. Thus in an *hfq*^*−*^ background there was an increase in the steady-state level of transposase transcript and this increase was slightly greater than that observed when methylation of the transposase promoter was blocked.

We also looked at the combined impact of knocking out Hfq and blocking DAM methylation on T1 levels (lanes 19 to 23 in Figure 5A). In comparison to *wt*, the ‘double mutant’ situation resulted in a 45-fold increase in T1 levels. Based on the observed synergy, we think it unlikely that the observed impact of deleting *hfq* is linked to the regulation of *dam* expression.

**Figure 6:**
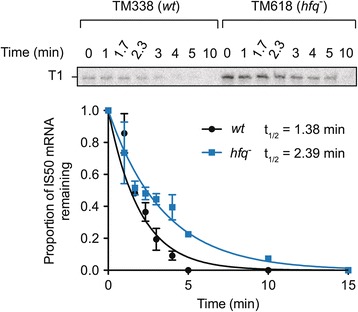
***IS50*** **transposase mRNA half-life analysis.** Strains TM338 (*wt*) and TM618 (*hfq* ^−^) were transformed with *IS50* transposase encoding plasmid pDH533 and total RNA was isolated either before or after the addition of rifampicin (at the indicated time points). Transposase RNA was detected as described in Figure 5. The bands were quantified (ImageQuant) and T1 normalized to un-extended primer before plotting the proportion of RNA remaining after rifampicin addition (time zero = 1.0). The data was fit to a one-phase exponential decay curve by non-linear regression (Prism) to determine the half-life (t_1/2_). The data shown is a compilation from two independent experiments.

To directly test if a component of Hfq-directed repression of *IS50* transposase expression is post-transcriptional, we compared the stability of the *IS50* transposase mRNA (T1) in isogenic *wt* and *hfq*^−^ strains. Total RNA was isolated from a pair of rifampicin-sensitive strains (TM338 and TM618) containing a plasmid encoding *IS50* transposase (pDH533) before and after rifampicin treatment as shown in Figure 6. Transposase mRNA was detected by primer extension. In the *hfq*^*−*^ strain the half-life of the T1 transcript increased by approximately 1.7-fold, revealing that *hfq* status does impact on transposase mRNA stability.

Taken together, the results from Figures 3, 4, 5 and 6 show that *IS50* transposase expression is substantially reduced in an *hfq*^*+*^ relative to an *hfq*^*−*^ strain and that *hfq* status primarily affects transposase transcription.

### Regulation of *Tn5* transposase expression by global transcriptional regulators

**Figure 7:**
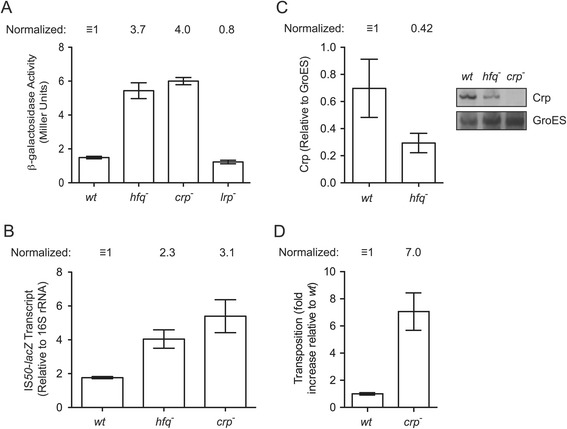
**Gene expression and** ***Tn5*** **transposition assays in strains harboring disruptions of global transcriptional regulators. (A)** β-galactosidase activity for isogenic strains (*wt*, *hfq* ^−^, *crp* ^−^ and *lrp* ^−^) harboring the TCF in single-copy in the chromosome (DBH303 and derivatives). Cells were grown to mid-log phase in Luria broth (LB). Mean and standard error values of duplicate experiments, each of which included at least three replicates, are shown. **(B)** *IS50*-*lacZ* transcript levels. Total RNA was extracted from cells described in panel **(A)**, and subjected to RT-PCR. **(C)** Western blot analysis of Crp levels in cellular extracts from *wt* and *hfq* ^−^ cells grown in LB. As a negative control, *crp* ^−^ cells were also analyzed. A representative image is shown in the inset. Crp levels were normalized to GroES, which is known to be insensitive to *hfq* status [19]. **(D)** *Tn5* transposition from the chromosome of DBH179 (wt) and DBH345 (crp^−^) was measured by the conjugal ‘mating out’ assay as described in Methods. The data is from a single experiment wherein five independent clones of each strain were tested. Mean and standard error values are shown. The average transposition frequency was 1.70 × 10^−4^ events per mL of mating mix for the *wt* strain and for purposes of comparison this value was set at 1 and the ‘*crp*’ value was normalized to this. In two other independent experiments the fold increase in *Tn5* transposition for *crp* ^*−*^ versus *wt* did not differ by more than 20% compared to the experiment shown (data not shown). For experiments in **(A-C)**, mean and standard error values from at least three independent isolates are shown.

As Hfq does not typically function directly in transcription, we set out to define a transcription factor that down-regulates *IS50* transposase transcription and is itself regulated by Hfq. Toward this end, we asked if disrupting genes for two global transcription factors, Crp and Lrp [18], had an impact on *IS50* transposase expression. Note that we had to construct new TCF reporter strains for this work because the available *crp* and *lrp* disruption strains we used to transduce DBH107 to either *crp*^*−*^ or *lrp*^*−*^ were marked with the same antibiotic resistance gene used to select for a lysogen with a chromosomal copy of the TCF. We show in Figure 7A that *crp*^−^ but not *lrp*^−^ had a substantial impact on transposase expression. For example, in cells grown in exponential phase in Luria broth (LB), there was up-regulation of transposase expression (approximately 4-fold) in both *crp*^*−*^ (DBH307) and *hfq*^*−*^ (DBH306) strains but not in the *lrp*^*−*^ strain (DBH315). We also performed semi-quantitative RT-PCR and show that transposase-*lacZ* transcript levels increased similarly in *crp*^*−*^ and *hfq*^*−*^ strains (Figure 7B). These results are consistent with Crp being a negative regulator of *IS50* transposase transcription.

We next asked if Crp expression was regulated by Hfq. Notably, work done in *Yersinia pestis* has shown that Hfq positively regulates Crp expression at the post-transcriptional level [20]. Towards this end we performed Western blot analysis with a Crp antibody on *E. coli* cell extracts from *wt* (DBH303), *hfq*^*−*^ (DBH306) and *crp*^*−*^ (DBH307) strains (Figure 7C). The results show that lower levels of Crp are present in the *hfq*^*−*^ strain, which is consistent with Hfq also being a positive regulator of Crp expression in *E. coli*.

Finally, we assessed the impact of knocking out *crp* on *Tn5* transposition frequency using the ‘mating out’ assay (Figure 7D). In the absence of *crp*, *Tn5* transposition increased 7-fold, which is consistent with results from the transposase expression experiments.

### *IS50* transposase expression and *Tn5* transposition are up-regulated by over-expression of the sRNA SgrS

**Figure 8:**
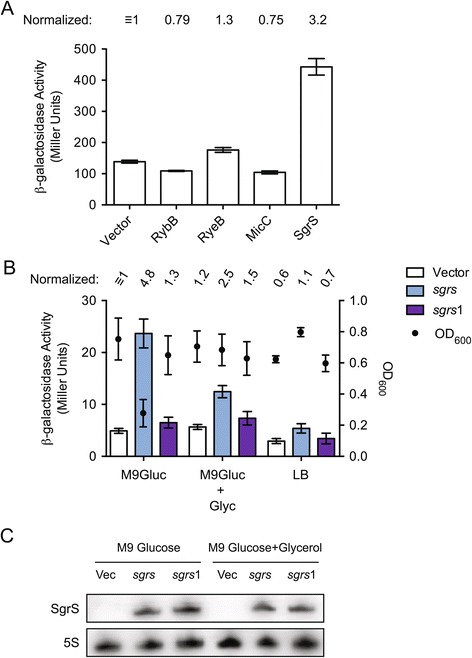
**Transposase-** ***lacZ*** **expression assays in cells over-expressing sRNAs. (A)** Transposase expression from an *IS50* translational fusion (TLF) (see Figure 3A) present on a low-copy plasmid (pDH798) was measured in the presence of a compatible plasmid expressing one of the indicated sRNAs from the inducible *pLlacO* promoter in DBH33. Cells were grown in M9 glucose and 0.1 mM IPTG was added to subcultures to induce sRNA expression. Transposase expression was measured 4 hours after IPTG addition. Expression levels were normalized to the strain with the vector only control. **(B)** The impact of different growth media on SgrS-induced up-regulation of transposase expression was evaluated using a single-copy TCF fusion (see Figure 3A) present in the chromosome of DBH265. Note that the *sgrS*1 allele of SgrS contains a two-nucleotide mutation that inhibits its ability to down-regulate expression of the glucose transporter encoded by *ptsG*. Subcultures were grown in either M9 glucose, M9 glucose + glycerol, or Luria broth (LB), as indicated. β-galactosidase activity was measured approximately 4 to 6 hours after subcultures were started. In **(A)** and **(B)** mean and standard error values of duplicate experiments, each of which included at least three replicates, are shown. **(C)** Northern blot of RNA isolated from cells in **(B)**. RNA was extracted from cells immediately before starting the Miller assay and visualized by Northern blotting with ^32^P-labeled RNA probes complementary to either SgrS or the 5S rRNA (internal control).

Over-expression of sRNAs can alter Hfq-regulated networks by limiting the availability of Hfq [21,22]. Given our findings that *Tn5* transposition and transposase gene expression are affected by *hfq* status, we asked if *IS50* transposase expression might be sensitive to Hfq-titration. Towards this end, we measured transposase expression from the TLF under conditions where a single sRNA was over-expressed from an IPTG inducible promoter (*pLlacO*) in DBH33, which is *lacI*^*q*^. Our initial screen included four different Hfq-dependent sRNAs, including RybB, RyeB, MicC and SgrS, all of which are expected to tightly bind Hfq *in vivo*; apparent equilibrium dissociation constants of approximately 3.3 nM and < 20 nM have been measured for MicC and SgrS, respectively [23-25]. Cells were grown in M9 glucose and sRNA expression was induced for 4 hours in exponential phase. We show in Figure 8A that only one of the sRNAs tested, SgrS, had a significant impact on transposase expression. Induction of SgrS increased transposase expression just over three-fold. Given the comparable Hfq binding affinities of the sRNAs tested, it seemed unlikely that SgrS expression was increasing transposase expression through an Hfq-titration mechanism.

SgrS down-regulates the expression of several known targets, including the primary glucose transporter encoded by the *ptsG* gene, a mannose transporter encoded by *manXYZ* and it up-regulates the expression of *yigL*, a phosphatase involved in phospho-sugar detoxification [26]. As we observed up-regulation of *IS50* transposase expression in cells over-expressing SgrS in M9 glucose media, we considered the possibility that this effect was a response to glucose limitation. In fact, we show in Additional file 2 that induction of SgrS in M9 glucose resulted in a substantial slowing of bacterial growth, as would be expected if nutrients had become growth-rate limiting. To further test the glucose limitation hypothesis, we performed a similar experiment in rich media (LB) and in M9 glucose supplemented with glycerol, a carbon source whose import is not dependent on glucose transporters [27]. We also tested the response of the reporter to over-expression of an SgrS mutant, *sgrS1*, that is incapable of down-regulating glucose import [28]. In these experiments we used a *Tn5* TCF as a reporter in the DBH107 strain background; DBH107 has a complete deletion of the *lac* operon and consequently the plasmid-encoded sRNA genes are constitutively expressed. To avoid problems in growing these cells, cultures were initially propagated in either LB or M9 glucose/glycerol and then where indicated, switched to other media.

We show in Figure 8B that after approximately 4 hours of SgrS over-expression in M9 glucose, reporter expression increased close to 5-fold relative to a ‘vector’ control. In contrast, over-expression of SgrS1 was incapable of up-regulating reporter expression under these same conditions, suggesting that SgrS must be able to down-regulate glucose import and or retention in order to increase transposase transcription. When cells were grown in M9 glucose supplemented with glycerol, expression of SgrS as above caused only an approximately 2-fold increase in transposase expression. Importantly, the reduced effects of SgrS on transposase expression under ‘glycerol’ conditions cannot be explained by differential expression of the respective sRNAs, as levels of SgrS and SgrS1 were similar in M9 glucose with or without glycerol (Figure 8C). Also, we failed to see significant transposase induction when SgrS was over-expressed in LB media where there are multiple carbon sources. Finally, consistent with the glucose limitation hypothesis, we also show in Figure 8B that increased transposase expression resulting from SgrS expression in M9 glucose was the only condition that inhibited cell growth.

**Figure 9:**
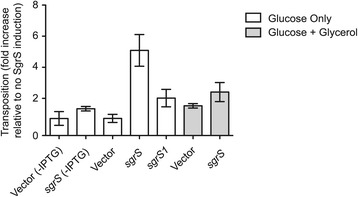
**Impact of SgrS expression on** ***Tn5*** **transposition.** Transposition of a chromosomal copy of *Tn5* was measured in DBH179 using the ‘mating out’ assay. DBH179 containing one of the indicated plasmids was grown overnight in M9 glucose and then subcultured in either M9 glucose or M9 glucose plus glycerol before mating with the recipient strain and plating on selective media as described in Figure 2. IPTG was added to the subculture (to 0.1 mM) to induce SgrS expression, except where indicated (-IPTG). The average transposition frequency for the ‘no SgrS’ control was 5.52 × 10^−5^ events per mL of mating mix. All other transposition frequencies were normalized to this value. Mean and standard error values of duplicate experiments, each of which included at least five replicates for each experimental group, are shown.

Given that transposition frequency is expected to be roughly proportional to transposase expression, we also asked if glucose limitation had an impact on *Tn5* transposition. Cells encoding a chromosomal copy of *Tn5* were transformed with an SgrS-expressing plasmid (or vector only control) and the frequency of *Tn5* transposition was measured using the ‘mating out’ assay. Note that cells were grown in M9 glucose media and SgrS expression was induced only when donor strains were subcultured on the day of mating. We show in Figure 9 that induction specifically of SgrS resulted in a 5-fold increase in *Tn5* transposition relative to the vector only control. Notably, when cells were grown in M9 supplemented with glucose and glycerol, induction of SgrS did not result in a significant increase in *Tn5* transposition. Also, we observed a reduced growth rate only in cultures where SgrS was induced in M9 glucose media (data not shown). The results of the ‘mating out’ analysis are thus entirely consistent with the gene expression experiments presented in Figure 8.

## Discussion

Hfq is a global regulator of gene expression in bacteria. However, until recently, Hfq had not been linked to the control of transposable elements. Work in the *Tn10*/*IS10* system provided the first example of Hfq inhibiting a transposon [7]. In the current work, we asked if the transposition of a related element, *Tn5*/*IS50*, is also regulated by Hfq. We show that *Tn5* transposition and *IS50* transposase expression are repressed by Hfq; however, the mechanism of repression is atypical for Hfq, involving predominantly a block in *IS50* transposase transcription. Preliminary evidence is presented that is consistent with Hfq modulating *IS50* transposase transcription through regulation of Crp. We also show that transposase transcription and *Tn5* transposition are activated by over-expression of the sRNA SgrS and provide evidence that this is a transcriptional response to glucose limitation.

### Hfq negatively regulates *Tn5* transposition

The results of ‘mating out’ experiments were consistent with Hfq acting as a strong negative regulator of *Tn5* transposition. *Tn5* transposition increased close to 75-fold in one *hfq*^*−*^ strain (DBH179 background). The magnitude of this increase was somewhat surprising given that up-regulation of *Tn10* in *hfq*^*−*^, under essentially antisense-minus conditions, was about 7-fold [7]. However, in a different *hfq*^*−*^ strain (DBH261 background) *Tn5* transposition increased only 9-fold. At this point it is unclear why there was such a large discrepancy in the ‘mating out’ values for the two strains. One possibility is that colony counts in the DBH179 ‘mating out’ (*hfq*^*−*^) included clones that had ‘jack-pot’ events. That is, colonies were counted that did not derive from independent transposition events. This could explain the high standard error associated with the transposition frequency in the *hfq*^*−*^ column in Figure 2A. If, for example, we removed the 3 most prominent outliers from the (DBH179) *hfq*^*−*^ data set, the fold increase in transposition dropped to 15-fold, which is more in line with what we observed in the DBH261 strain background and for *Tn10* in single copy [7].

A trans-complementation (Figure 2A) experiment provided definitive proof that the increase in *Tn5* transposition detected in one of our *hfq*^*−*^ ‘mating out’ strains (DBH179 background) was in fact due to *hfq* deficiency. In addition, the failure of two Hfq RNA-binding face mutants to provide complementation was consistent with Hfq-directed inhibition of *Tn5* transposition relying on functions of Hfq required in canonical Hfq-directed regulatory pathways [17]. That is, Hfq must retain the ability to bind both mRNAs and sRNAs to influence *Tn5* transposition.

### Hfq, Crp and *IS50* transposase gene expression

Evidence that *hfq* status influences *IS50* transposase expression came from two types of experiments. First, the expression of transposase-*lacZ* reporter genes in both transcriptional and translational fusion constructs increased significantly under conditions of *hfq* deficiency. Second, the steady-state level of the native transposase transcript also increased significantly in *hfq*^*−*^. Importantly, the large increase in steady-state transcript level (11-fold) coincided with a less substantial increase in transposase mRNA stability (less than 2-fold increase in half-life). In addition, up-regulation of reporter expression in *hfq*^*−*^ for a TLF was almost completely abrogated when the *IS50* transposase promoter was replaced by a heterologous promoter. Taken together, these results are consistent with Hfq (or a factor regulated by Hfq) suppressing *IS50* transposase expression predominantly at the level of transcription. Notably the suppressive effect of Hfq on *IS50* transposase transcription was remarkably specific, as the level of a second transcript (T2) encoded by *IS50* was not affected by *hfq* status.

As Hfq does not typically act directly in gene transcription, we think it likely that Hfq acts indirectly on the *IS50* transposase promoter. In addition to DAM, only one other transcription factor, LexA, has been implicated as a regulator of transposase transcription. There is a weak LexA-binding site in the transposase promoter (Figure 1A); however, *lexA* deficiency was shown to increase transposase transcription only two to three-fold in a TCF [16]. As we have seen increases in transposase expression of up to 11-fold for a TCF in *hfq*^−^, it seems unlikely that Hfq would be working through LexA. In contrast, transposase expression increased in *dam*^*−*^ to a level more in line with that observed in *hfq*^*−*^ (less than two-fold difference in the TCF). However, the observed synergy between *hfq*^*−*^ and mutations that rendered the *IS50* transposase promoter DAM-insensitive led us to conclude that Hfq does not regulate *IS50* transcription by impacting DAM levels (and, therefore, promoter methylation). These results provided motivation to search for other targets of Hfq that impinge on *IS50* transposase transcription. This search identified Crp as an additional negative regulator of *IS50* transposase transcription. Notably, transposase expression increased to approximately the same level in *crp*^*−*^ and *hfq*^*−*^ in the experiment in Figure 7. The similar magnitude of up-regulation of transposase expression in *hfq*^−^ and *crp*^−^ could be indicative of Hfq acting upstream of Crp to inhibit transposase expression. We did in fact find evidence of Hfq positively regulating Crp protein levels (Figure 7C). This observation is consistent with work recently published in the *Y. pestis* system where it was found that Crp protein levels decreased approximately five-fold in an *hfq* disruption strain [20].

Crp is a known activator/repressor of transcription [18] and, therefore, more likely than Hfq to be directly involved in regulating *IS50* transposase expression at the transcriptional level. Given our evidence that Hfq positively regulates *crp* expression, a plausible scenario explaining our expression data is that the observed up-regulation of *IS50* transposase transcription in *hfq*^*−*^ is a result of decreased Crp protein levels. Crp may act either directly or indirectly on the *IS50* transposase promoter to repress transcription. This is currently a working model as we have not yet tested the possibility that Crp binds the *IS50* transposase promoter and it may only be coincidental that transposase expression increased to similar levels in *hfq*^*−*^ and *crp*^*−*^ strains. Notably, we also found that *Tn5* transposition increased when the *crp* gene was disrupted, although the extent of the increase was smaller than that observed in the isogenic *hfq* disruption strain. This could be indicative of additional factors in the Hfq regulon impinging on *Tn5* transposition.

There is precedent for Crp down-regulating the transcription of a transposase gene. In the case of *IS2*, transposase transcription increased close to 200-fold in *crp*^*−*^. It was also shown through protein-DNA footprinting that Crp binds directly to the *IS2* transposase promoter [29]. Interestingly, based on the consensus binding sequence for Crp, the authors of the above study predicted that Crp would bind to the *IS50* transposase gene. However, the predicted *crp* binding site is located downstream of the transposase promoter and is not present in our TCF (where we detected increased transposase expression in *crp*^*−*^). Nevertheless, it would be worthwhile to test for Crp binding to the *IS50* transposase promoter as the results of Crp ChIP-chip studies revealed the presence of thousands of weak *crp* binding sites scattered throughout the *E. coli* genome [30]. It is also possible that Crp acts indirectly on the *IS50* transposase promoter by regulating the expression of another transcription factor.

### *Tn5* transposition and metabolic stress

We also identified conditions that activate transposase expression and transposition; over-expression of the sRNA SgrS increased transposase expression and transposition approximately five-fold. We favor the possibility that this induction is a consequence of glucose limitation but cannot formally rule out the possibility that SgrS targets an as yet undefined regulatory pathway that impinges on transposase expression. Our reasoning for this is that we observed induction of transposase expression and transposition specifically when cells were grown with glucose as the major carbon source and SgrS is known to prevent expression and function of the major glucose transporter encoded by the *ptsG* gene [26]. Consistent with this idea, we found that transposase induction levels correlated with a reduced growth rate. Furthermore, we demonstrated that: (i) an allele of SgrS (*sgrS1*) that is incapable of down-regulating *ptsG* expression failed to induce transposase expression in M9 glucose; (ii) under conditions where SgrS was expressed in M9 glucose media supplemented with glycerol, we failed to see induction of transposase expression to the same extent as when glycerol was absent; (iii) SgrS expression did not impact transposase expression when cells were grown in rich media (LB) and (iv) over-expression of 3 other sRNAs (RybB, RyeB and MicC) that are not expected to influence glucose transport did not increase transposase expression in M9 glucose [31-33]. Precedent for nutritional stress influencing transposition comes from earlier work in the *IS903* system where mutations in a gene (*aspA*) required for fermentative metabolism during anaerobic growth caused transposition to occur at an accelerated rate [34].

At this point it is unclear as to what factors are driving the induction of the transposase gene under SgrS over-expression conditions. With regard to further defining the mechanism of *IS50* transposase up-regulation under SgrS over-expression conditions, it would also be advantageous to find alternative experimental conditions for achieving this increased expression. If, for example, simply starving cells by restricting a carbon source during growth achieves the same end as over-expressing SgrS in M9 glucose media, an unbiased screen to search for genetic factors that are necessary for the up-regulation of transposase expression could be performed to reveal the regulatory network impinging on the transposase promoter. As it stands, any factors that influence SgrS expression would interfere with the outcome of such a screen. Alternatively, if it was found that restricting glucose is not sufficient for inducing transposase expression, the possibility that SgrS plays a more direct role in controlling transposase expression would have to be considered.

## Conclusions

In this work, we have identified several genes that impact on *IS50* transposase expression, including *hfq*, *crp* and *sgrS*. Hfq and Crp proteins are negative regulators and SgrS RNA (under specific growth conditions) is a positive regulator of transposase gene expression. Exactly how these factors impinge on transposase expression remains to be worked out and at this point it is not clear if we are seeing modulation of the same regulatory network in opposite directions when *hfq* and *crp* genes are disrupted and SgrS RNA is over-expressed. *Tn5*/*IS50* is the second transposon identified that is affected by disruption of the *hfq* gene and the first that does not encode an antisense RNA. This raises the possibility that Hfq influences the transposition frequency of many other bacterial transposons.

## Methods

### Plasmids, bacteriophage and strains

The *IS50* translational fusion plasmid (pDH798) is a pWKS30-derivative containing base pairs 1 to 431 of *IS50* (nucleotides 1 to 366 of T1) fused to codon 10 [35] of the *E. coli lacZ* gene. The *IS50* transcriptional fusion plasmid (pDH682) is a pUC18-derivative containing base pairs 1 to 80 of *IS50* (nucleotides 1 to 15 of T1) fused to nucleotide -16 (relative to the translational start codon) of *lacZ*. Plasmids encoding sRNAs (pDH764, *sgrS*; pDH766, *rybB*; pDH768, *micC*; pDH772, *ryeB*) and the corresponding empty vector control (pDH763) were kindly provided by S Gottesman. The plasmid encoding *sgrS1* (pDH895) was kindly provided by C Vanderpool. Plasmids encoding Hfq (pDH700, *wt*) and mutant derivatives (pDH701, K56A; pDH713, Y25A) are described in Ross *et al* [8]. Details of plasmid constructions are provided in Additional file 3 and a list of oligonucleotides used in this work is provided in Additional file 4.

Lambda phages encoding *IS50* transcriptional (λDBH849 and λDBH888) and translational (λDBH812) reporters were generated by cloning *IS50* expression cassettes marked with an antibiotic resistance gene (either *kan*^R^ or *cm*^R^) into the *his* operon of pNK81 and then infecting a strain harboring one of these plasmids with λNK1039, which also contains the *his* operon. Antibiotic resistant lysogens from the above crosses were selected by replica plating and subsequently phage released from the lysogens were purified, giving rise to λDBH849 (*IS50*-*lacZ*-*kan*^R^ TCF), λDBH888 (*IS50-lacZ-Cm*^R^ TCF) and λDBH812 (*IS50-lacZ-Kan*^R^ TLF).

*E. coli* strains for the ‘mating out’ assay were constructed by P1 transduction of *Tn5* from ER2507 (NEB) into DBH33, DBH344 and DBH259. Strains containing chromosomal *IS50*-*lacZ* fusions were generated by lysogenizing DBH107 with λDBH849 (DBH265), λDBH888 (DBH303) or λDBH812 (DBH281). Mutant derivatives of these strains were generated by P1 transduction. A list of all of the strains, plasmids and bacteriophage used in this work is presented in Table 1.

**Table 1:** **Plasmids, bacteriophage and strains**

**Strain or Plasmid**	**Relevant genotype**	**Use**	**Source or reference**
***E. coli***			
DBH13	HB101 [F^−^ *leu* ^−^ *pro* ^−^]; Str^R^	‘Mating out’ recipient	[36]
ER2507	*zjc::Tn5*; Kan^R^	Source of *zjc::Tn5*	NEB
DBH179	NK5830 [*recA* ^−^ *arg* ^−^/F’ *lacpro* ^+^] *zjc::Tn5*; Kan^R^	‘Mating out’ donor	This study
DBH184	DBH179 *hfq*-1::Ωcat; Kan^R^Cm^R^	‘Mating out’ donor	This study
DBH228	RZ211/pOX38Gen	Source of pOX38Gen	[37]
DBH233	HW-5 [*phoA4*(Am) *his-45 recA1 rpsL99 met-54* F^−^]; Str^R^	Parent strain	[38]
DBH259	DBH233/pOX38Gen; Str^R^Gen^R^	Parent strain	This study
DBH261	DBH259 *zjc::Tn5*; Str^R^Gen^R^Kan^R^	‘Mating out’ donor	This study
DBH271	DBH261 *hfq*-1::Ωcat; Str^R^Gen^R^Kan^R^Cm^R^	‘Mating out’ donor	This study
DBH272	DBH261 *dam*::Tn9cat; Str^R^Gen^R^Kan^R^Cm^R^	‘Mating out’ donor	This study
DBH107	MC4100 [F^−^ Δ(argF-lac)169* rpsL150]; Str^R^	Parent strain	[39]
DBH265	DBH107/λDBH849; Str^R^Kan^R^	Miller Assay	This study
DBH267	DBH265 *hfq*-1::Ωcat; Str^R^Cm^R^Kan^R^	Miller Assay	This study
DBH268	DBH265 *dam*::Tn9cat; Str^R^Cm^R^Kan^R^	Miller Assay	This study
DBH281	DBH107/λDBH812; Str^R^Kan^R^	Miller Assay	This study
DBH283	DBH281 *hfq-1*::Ωcat; Str^R^Cm^R^Kan^R^	Miller Assay	This study
DBH285	DBH281 *dam*::Tn9cat; Str^R^Cm^R^Kan^R^	Miller Assay	This study
DBH303	DBH107/λDBH888; Str^R^Cm^R^	Miller Assay	This study
DBH306	DBH303 Δ*hfq*722::kan; Str^R^Cm^R^Kan^R^	Miller Assay	This study
DBH307	DBH303 Δ*crp*765::kan; Str^R^Cm^R^Kan^R^	Miller Assay	This study
DBH315	DBH303 Δ*lrp*787::kan; Str^R^Cm^R^Kan^R^	Miller Assay	This study
DBH33	NK5830 [*recA* ^−^ *arg* ^−^/F’ *lacpro* ^+^]	Parent strain	[40]
DBH16	DBH33 *hfq*-1::Ωcat; Cm^R^	Parent strain	[7]
DBH241	DBH33 *dam*::Tn9cat; Cm^R^	Parent strain	This study
DBH238	DBH33/λDBH849; Kan^R^	Miller Assay	This study
DBH239	DBH238 *hfq*-1::Ωcat; Kan^R^Cm^R^	Miller Assay	This study
DBH240	DBH238 *dam*::Tn9cat; Kan^R^Cm^R^	Miller Assay	This study
DBH208	DBH33/λDBH812; Kan^R^	Miller Assay	This study
DBH210	DBH208 *hfq*-1::Ωcat; Kan^R^Cm^R^	Miller Assay	This study
DBH237	DBH208 *dam*::Tn9cat; Kan^R^Cm^R^	Miller Assay	This study
DBH323	DBH107 *recA* ^−^; Str^R^	Miller Assay	This study
DBH326	DBH107 *recA* ^−^ *hfq*-1::Ωcat; Str^R^Cm^R^	Miller Assay	This study
DBH242	DBH33 Δ*crp*765::kan	Parent strain	This study
DBH344	DBH242 Δ*crp*765; Kan^S^	Parent strain	This study
DBH345	DBH344 *zjc::Tn5; Kan* ^*R*^	‘Mating out’ donor	This study
TM338	W3110*mlc rne-Flag-cat*; rif^S^Cm^R^	RNA half-life measurements	[41]
TM618	W3110*mlc rne-Flag-cat* Δ*hfq*; rif^S^Cm^R^	RNA half-life measurements	[42]
DH5α	*recA* ^−^	Plasmid propagation	Invitrogen
**Plasmids**			
pWKS30	pSC101-derived; low copy-number ori ; Ap^R^	‘Empty vector’ for Hfq expression	[35]
pDH700	pWKS30-P3-*hfq*_*WT*_; Ap^R^	Hfq_WT_ expression	[7]
pDH701	pWKS30-P3-*hfq*_*K56A*_; Ap^R^	Hfq_K56A_ expression	[7]
pDH713	pWKS30-P3-*hfq*_*Y25A*_; Ap^R^	Hfq_Y25A_ expression	[8]
pDH533	pUC18-derivative; *Tn5* t’ase M56A; Ap^R^Cm^R^	Source of *Tn5* transposase (No Inh.)	[43]
pDH752	pDH533 with t’ase mutated to G53A,C61A; Ap^R^Cm^R^	DAM-insensitive t’ase	This study
pDH828	pDH533 with t’ase mutated to D97A; Ap^R^Cm^R^	Catalytic^−^ t’ase	This study
pNK81	pBR333-derivative; encodes his operon; Ap^R^	Lambda crosses	[44]
pDH682	pUC18-derivative; *IS50*-*lacZ* TCF; Ap^R^	Source of TCF	This study
pDH838	pDH682-derivative; TCF ‘marked’ with kan^R^	Parent of pDH849	This study
pDH883	pDH682-derivative; TCF ‘marked’ with cm^R^	Parent of pDH888	This study
pDH849	TCF-kan^R^ from pDH682 cloned into BclI-cut pNK81; Ap^R^Kan^R^	For crossing TCF onto λ	This study
pDH888	TCF-cm^R^ cloned onto BclI-cut pNK81; Ap^R^Cm^R^	For crossing TCF onto λ	This study
pDH658	pRZ9905-derivative; full-length *IS50*-*lacZ* TLF; Ap^R^	Parent of pDH795	This study
pDH795	pDH658-derivative; ‘deletion’ TLF used in this study; Ap^R^	Parent of pDH804	This study
pDH804	pDH795-derivative; TLF ‘marked’ with kan^R^	Parent of pDH812	This study
pDH812	TLF-kan^R^ cloned into BclI-cut pNK81; Ap^R^Kan^R^	For crossing TLF onto λ	This study
pDH753	pWKS30-derivative; contains *IS50*-*lacZ* TLF from pDH658; Ap^R^	Parent of pDH798	This study
pDH798	pDH753-derivative; Ap^S^Kan^R^	Miller Assay	This study
pDH763	pBR-*plac*; Ap^R^	Vector for sRNA-induction	[45]
pDH764	pBR-*plac-sgrS*; Ap^R^	SgrS-induction	[46]
pDH895	pBR-*plac-sgrS1*; Ap^R^	SgrS1-induction	[47]
pDH766	pBR-*plac-rybB*; Ap^R^	RybB-induction	[48]
pDH768	pBR-*plac-micC*; Ap^R^	MicC-induction	[48]
pDH772	pBR-*plac-ryeB*; Ap^R^	RyeB-induction	[48]
pDH908	pDH795-derivative; Lpp-TLF	Miller Assay	This study
**Phage**			
λNK1039	Encodes his operon	Parent phage	[49]
λDBH812	*IS50*-*lacZ* translational fusion (TLF) from pDH812 marked with kan^R^	Chromosomal TLF construction	This study
λDBH849	*IS50*-*lacZ* transcriptional fusion (TCF) marked with kan^R^	Chromosomal TCF construction	This study
λDBH888	*IS50*-*lacZ* transcriptional fusion (TCF) marked with cm^R^	Chromosomal TCF construction	This study

### ‘Mating out’ assay

Conjugal ‘mating out’ experiments were performed essentially as described for single-copy chromosomal transposons in Ross *et al*. [7], except that for measuring transposition in *hfq*^*−*^ versus *wt,* donor growth was carried out in M9 glucose media supplemented with kanamycin (25 μg/mL) and amino acids, instead of LB. DBH13 was used as the recipient. Total exconjugants and transposition events with DBH179 and derivatives were scored by plating mating mixes on M9 glucose plates supplemented with leucine, thiamine and streptomycin (150 μg/mL) or streptomycin and kanamycin (25 μg/mL), respectively. Total exconjugants and transposition events with DBH261 and derivatives were scored by plating mating mixes on M9 glucose plates supplemented with leucine, thiamine, streptomycin (150 μg/mL) and gentamicin (12.5 μg/mL) or streptomycin, gentamicin and kanamycin (25 μg/mL), respectively.

### β-galactosidase assays

Cells were grown in M9 glucose (with arginine and thiamine) or LB. In situations where strains contained plasmids, plasmids were maintained by including the appropriate antibiotic. Overnight cultures (0.05 mL) were used to seed subcultures (1.5 mL), which typically were grown to mid-log phase before being processed for the Miller assay as previously described [7].

### RNA isolation, primer extension and Northern blot analysis

Total RNA was isolated essentially as described in [50]. For steady-state analysis, cells were grown to mid-log phase in LB before RNA isolation. For half-life analysis, rifampicin (dissolved in dimethyl sulfoxide; DMSO) was added to cell cultures (to 200 μg/mL) to arrest transcription and RNA was isolated immediately before and after rifampicin addition at the indicated time intervals. Primer extension analysis was carried out using ^32^P-labeled primers oDH230 and oDH390, end-labeled with OptiKinase (USB, Cleveland, OH, USA) according to manufacturer’s instructions. Extension reactions used 5 μg of RNA, and Superscript III reverse transcriptase essentially as described in [51], except that annealing was performed at 65°C (with no ice treatment) before extending at 55°C for 45 minutes. Extension products were resolved on 6% and 10% denaturing polyacrylamide gels. For Northern blot analysis, 2 μg of RNA was mixed with an equal volume of denaturing load dye (95% deionized formamide [v/v], 10 mM EDTA, 0.5× TBE, 3% xylene cyanol [w/v]), heated to 95°C for 2 minutes, and resolved on a 6% polyacrylamide gel containing 7 M urea. Separated RNAs were electro-transferred to Hybond N (GE Healthcare, Mississauga, ON, Canada) in 0.5× TBE and fixed with UV. Annealing and washing was performed in ULTRAhyb buffer (Ambion, Burlington, ON, Canada) according to the manufacturer’s instructions, using RNA probes complimentary to SgrS or the 5S rRNA (internal standard). To construct the radiolabeled RNA probes, DNA templates for *in vitro* transcription were made by PCR with primers oDH232/233 (SgrS) and oDH234/235 (5S rRNA) - note that, for each primer pair, the forward primer includes the T7 core promoter. These templates were transcribed *in vitro* in the presence of ^32^P-UTP to generate uniformly labeled RNA probes. *In vitro* transcription reactions were performed in 25 μL volumes with approximately 1 μg DNA template, 1 × T7 RNA polymerase buffer (NEB, Beverly, MA, USA), 20 units RNasin (Promega, Madison, WI, USA), 4 mM dithiothreitol (DTT), 0.16 mg/mL BSA, 0.4 mM each of GTP, CTP and ATP, 0.01 mM UTP, 50 μCi [α-^32^P]UTP, and 100 units of T7 RNA polymerase.

### Western blot

Cells were centrifuged (2 minutes at 21,000 × *g*), resuspended in SDS load mix (2% [w/v] SDS, 10% [v/v] glycerol, 50 mM Tris-HCl pH 6.8, 0.25% [w/v] bromophenol blue, 0.8 M β-mercaptoethanol) and heated at 95°C for 5 minutes. To normalize for differences in growth between the various samples, the OD_600_ of each sample was measured and the volume spun normalized to give an equivalent to OD_600_ approximately equal to 0.35. The resulting lysates were subjected to SDS-PAGE on a 12% polyacrylamide gel, proteins transferred to PVDF (Roche, Indianapolis, IN, USA) and Crp was detected by Western blot with a polyclonal rabbit anti-Crp antibody (kind gift of H Aiba). The primary antibody was diluted 1:20,000 in TBST; the secondary antibody (anti-rabbit IgG-horseradish peroxidase (HRP) conjugate; Promega, Madison, WI, USA) was used at 1:5,000. Crp was visualized with a Pierce ECL 2 Western blotting substrate (Thermo Scientific, Rockford, IL, USA) and PhosphorImager (GE Healthcare). The membranes were stripped and GroES detected (rabbit anti-GroES antibody from Sigma-Aldrich (St Louis, MO, USA) at 1:10,000) for use as an internal standard; GroES is not sensitive to *hfq* status [19]. Bands were quantified using ImageQuant software (GE Healthcare) and Crp levels plotted relative to GroES.

## Additional files

Additional file 1:
**Mapping** ***Tn5*** **transposition events.** Southern blot and ST-PCR characterization of *Tn5* transposition events in *wt* and *hfq* ^*−*^ strains. Additional file 2:
**Impact of SgrS over-expression on growth rate in M9 glucose.** Growth curves of cells in which SgrS RNA was or was not induced by IPTG addition and corresponding Northern blot showing SgrS levels. Additional file 3:
**Details of plasmids constructed for this work.**
Additional file 4:
**List of oligonucleotides used in this work.**

## Abbreviations

BSA: bovine serum albumin
Crp: cyclic AMP-receptor protein
DAM: DNA adenine methylase
DMSO: dimethyl sulfoxide
DTT: dithiothreitol
EDTA: ethylenediaminetetraacetic acid
LB: Luria broth
PAGE: polyacrylamide gel electrophoresis
PCR: polymerase chain reaction
RT-PCR: reverse transcription polymerase chain reaction
sRNA: small RNA
SDS: sodium dodecyl sulfate
TBE: Tris borate-EDTA buffer
TBST: 20 mM Tris-HCl (pH 7.5), 150 mM sodium chloride, 0.5% Tween-20
TCF: transcriptional fusion
TIR: translation initiation region
TLF: translational fusion
UTR: untranslated region
UV: ultraviolet light
