# Supplementary material for: Tn5 transposition in Escherichia coli is repressed by Hfq and activated by over-expression of the small non-coding RNA SgrS
Source: Mob DNA. 2014 Nov 30;5:27. doi: 10.1186/s13100-014-0027-z (PMC4265352; doi:10.1186/s13100-014-0027-z)
Supplement: Additional file 1: — Mapping Tn5 transposition events. Southern blot and ST-PCR characterization of Tn5 transposition events in wt and hfq − strains. [file 13100_2014_27_MOESM1_ESM.docx]

**Additional File 1. Mapping Tn5 transposition events**

**A. Southern blot analysis of colonies selected on ‘hop plates’**

Genomic DNA was isolated from Kan^R^ Sm^R^ colonies selected on ‘hop plates’ using Sigma Genomic DNA kit; 1.5 μg of genomic DNA was digested with AccI (cuts once in Tn*5* in the *kan*^R^ gene) and resolved on a 1% agarose gel, before capillary transfer to Hybond N membrane (GE Healthcare) as follows: the gel was soaked in alkaline denaturing buffer (1.5 M NaCl, 0.5 M NaOH) for 40 minutes, neutralized (30 minutes and then 15 minutes) in neutralizing buffer (250 mM Tris-HCl, pH 7.5, 750 mM NaCl), and transferred overnight in 10x SSC. The membrane was UV cross-linked and then incubated with a ^32^P-labeled RNA probe (complementary to the 5’ portion of the Tn*5* *kan*^R^ gene) in UltraHyb buffer (Ambion) according to the manufacturer’s protocol for RNA:DNA hybridization. The probe was generated by *in vitro* transcription, as described for Northern blot analysis, from a DNA template made by PCR with primers oDH236 and oDH237. In the experiment shown 5 ‘hop’ colonies from each of *hfq^+^* (DBH179) and *hfq^-^* (DBH184) were analyzed. ‘+’ indicates DNA derived from a donor colony before mating and ‘-’ indicates DNA derived from a recipient colony before mating.

**B. Mapping Tn*5* tranposition events via ST-PCR**

Putative transposition events from mating out assays were characterized using a semi-random, two-step PCR protocol (ST-PCR) for uncharacterized transposon-linked sequences (Chun et al, 1997). Briefly, genomic DNA from Kan^R^ Sm^R^ colonies selected on ‘hop plates’ was PCR-amplified with a Tn*5*-specific primer (oDH225) and a partially randomized primer (oDH167). The resulting amplicons were ligated into the pGEM T-easy vector (Promega), transformed into DH5α by electroporation and transformants were selected on LB plates containing kanamycin (50 μg/mL). Plasmid DNA was isolated (Sigma miniprep kit) and the inserts were sequenced using M13 primers. Sequences thusly obtained were aligned to the *E. coli* MG1655 reference genome (BLAST) to identify the ‘target’ sequence immediately flanking one host-OE junction. This information was used to design ‘insert-specific’ primers (different for each insertion event), complementary to DNA flanking the Tn*5* insertion site. These primers were used to amplify and sequence DNA flanking Tn*5*.

**C. DNA sequence for two independent Tn5 transposition events**

Tn5 insertion sites for two clones are shown. Tn5 sequence is in red and flanking donor sequence is in black. Target site duplications are underlined.
