# Supplementary material for: Tn5 transposition in Escherichia coli is repressed by Hfq and activated by over-expression of the small non-coding RNA SgrS
Source: Mob DNA. 2014 Nov 30;5:27. doi: 10.1186/s13100-014-0027-z (PMC4265352; doi:10.1186/s13100-014-0027-z)
Supplement: Additional file 2: — Impact of SgrS over-expression on growth rate in M9 glucose. Growth curves of cells in which SgrS RNA was or was not induced by IPTG addition and corresponding Northern blot showing SgrS levels. [file 13100_2014_27_MOESM2_ESM.docx]

**Additional File 2. Impact of SgrS over-expression on growth rate in M9-glucose**

**A. Growth curve for cells harboring the SgrS-encoding plasmid with and without IPTG treatment.** A saturated culture of cells (DBH33) harboring the SgrS-overexpression plasmid (pDH764) was sub-cultured (in duplicate) into M9-glucose and grown for 4 hours. At this time IPTG (0.1 mM) was added to one of the sub-cultures. Cell density (OD_600_) was measured at the indicated time points.

**B. Measurement of SgrS RNA levels.** SgrS RNA levels were measured in cells from (A) at the indicated times after sub-culture by performing Northern blot analysis with ^32^P-labeled RNA probes complementary to SgrS or 5S rRNA (internal standard). Northern blot analysis was performed as described in Figure 8C.
