# Supplementary material for: Tn5 transposition in Escherichia coli is repressed by Hfq and activated by over-expression of the small non-coding RNA SgrS
Source: Mob DNA. 2014 Nov 30;5:27. doi: 10.1186/s13100-014-0027-z (PMC4265352; doi:10.1186/s13100-014-0027-z)
Supplement: Additional file 3: — Details of plasmids constructed for this work. [file 13100_2014_27_MOESM3_ESM.docx]

**Additional File 3. Details of plasmids constructed for this work**

*IS50 transposase expression plasmids:* Plasmids for *in vivo* expression of transposase from its native promoter are derivatives of pRZ9905 [1], obtained from W.S. Reznikoff. The transposase gene has the M56A mutation, which blocks translation of the inhibitor protein. In addition, a mini-Tn*5* encoding the chloramphenicol resistance gene from IS*1* was cloned into the BglII site just downstream from the transposase stop codon to create pDH533. Mutant forms of this plasmid including cat^-^ transposase (D97A — pDH828) and Dam-Insen transposase (G53A; C61A — pDH752) were generated by site-directed mutagenesis. We used the mini-Tn*5* element to test the functionality of various transposase-producing genes.

*IS50-lacZ translational fusion plasmids:* A PCR fragment containing a portion of the *lacZ* gene (starting at codon 10 and terminating at the stop codon) was generated with primers oDH185 and oDH186 and *E. coli* genomic DNA. Primer oDH185 includes a SacII site and oDH186 includes a BglII site. After digestion with SacII and BglII, the *lacZ* PCR fragment was cloned into pRZ9905, which had also been digested with SacII and BglII, generating pDH658. The IS*50* translational fusion from pDH658 was also cloned on an XmnI-SalI fragment into XmnI-SalI digested pWKS30 [2] to create pDH753. The IS*50* transposase coding sequence in pDH658 (includes up to codon 122 of transposase) was further truncated to include only the first 12 codons of transposase by digesting pDH658 with XmnI and SacII and cloning a PCR fragment generated with primers oDH187 and oDH188 (and cut with XmnI and SacII) into the aforementioned backbone to generate pDH795. The IS*50*-*lacZ* translational fusions pDH753 and pDH795 were linked to a kanamycin resistance gene from IS*903* (generated by PCR using primers oDH189 and oDH190) by cloning the *kan*^R^ gene present on a SpeI-XbaI cut PCR fragment into the XbaI site just downstream of the *lacZ* gene to create pDH798 and pDH804, respectively. The IS*50-lacZ-kan*^R^ fragment in pDH804 was PCR amplified with primers oDH191 and oDH192, each of which includes a BamHI site, and cloned into the large fragment (~ 6 kb) of pNK81 [3] generated by BclI digestion, creating pDH812. In this context, the IS*50* transposase-*lacZ-kan*^R^ fragment is flanked by *his* operon sequences and can be crossed onto λNK1039 [4], which also contains *his* operon sequences. A derivative of pDH795 in which the *IS50* transposase promoter was replaced with the promoter of the *lpp* gene (pDH908) was constructed by cloning a PCR fragment amplified with a forward primer (oDH331) containing the *lpp* promoter linked to *IS50* sequence (corresponding to the +1 position of the transposase transcript) and a reverse primer (oDH332) overlapping the *IS50*-*lacZ* junction sequence into EcoRI-SacII cut pDH795.

*IS50-lacZ transcriptional fusion plasmids:* A PCR fragment including the 5’UTR to the stop codon of the *lacZ* gene, generated with primers oDH193 (includes an AflII site) and oDH194 (includes a BamHI site) was cloned into pRZ9905, which had also been cut with AflII and BamHI, to create pDH682. The IS*50-lacZ* transcriptional fusion in pDH682 was then linked to either the *kan*^R^ gene of IS*903* (pDH838) or the *cm*^R^ gene of pACYC184 (pDH883); the latter was generated with primers oDH195 and oDH196, each of which contains an XbaI site. The IS*50* transposase-*lacZ-kan*^R^ and the IS*50* transposase-*lacZ-cm*^R^ segments of pDH838 and pDH883 were then PCR amplified using primers oDH191 and oDH192 and, each of which contains a BamHI site, and cloned into BclI-digested pNK81 as previously described, generating pDH849 and pDH888. The latter plasmids were used to cross the transcriptional fusions onto λNK1039.

**References**

1. Naumann TA, Reznikoff WS: **Trans catalysis in Tn5 transposition**. *Proceedings of the National Academy of Sciences of the United States of America* 2000, **97**(16):8944-8949.

2. Wang RF, Kushner SR: **Construction of versatile low-copy-number vectors for cloning, sequencing and gene expression in Escherichia coli**. *Gene* 1991, **100**:195-199.

3. Foster TJ, Davis MA, Roberts DE, Takeshita K, Kleckner N: **Genetic organization of transposon Tn10**. *Cell* 1981, **23**(1):201-213.

4. Bender J, Kuo J, Kleckner N: **Genetic evidence against intramolecular rejoining of the donor DNA molecule following IS10 transposition**. *Genetics* 1991, **128**(4):687-694.
