# Supplementary material for: Tn5 transposition in Escherichia coli is repressed by Hfq and activated by over-expression of the small non-coding RNA SgrS
Source: Mob DNA. 2014 Nov 30;5:27. doi: 10.1186/s13100-014-0027-z (PMC4265352; doi:10.1186/s13100-014-0027-z)
Supplement: Additional file 4: — List of oligonucleotides used in this work. [file 13100_2014_27_MOESM4_ESM.docx]

**Additional File 4. List of oligonucleotides used in this study**

| Name | Sequence (5’ to 3’) | Use |
| --- | --- | --- |
| oDH167  oDH183  oDH184  oDH185  oDH186  oDH187  oDH188  oDH189  oDH190  oDH191  oDH192  oDH193  oDH194  oDH195  oDH196  oDH204  oDH205  oDH225  oDH230  oDH232  oDH233  oDH234  oDH235  oDH236  oDH237  oDH238  oDH239  oDH240  oDH331  oDH332  oDH390 | GGCCACGCGTCGACTAGTACNNNNNNNNNNGATAT  NNAAGCTTNNTTATTCGGTTTCTTCGCT  NNTCTAGANNCAGGTTGTTGGTGCTATC  NNNCCGCGGAGTCGTTTTACAACGTCGTGACTGGGAAAACCC  NNNAGATCTTTATTTTTGACACCAGACCAACTGGTAATGGTAGCG  CATCATTGGAAAACGTTCTTCGGGGCG  AACGACTCCGCGGCTGTCGGCCGCACGATGAAGAGC  GGACTAGTGCCAGTGTTACAACCAATTAACCAATTCTGA  GCTCTAGACTGAGAGATCCCCTCATAATTTCCCCTCAGCAAAAGTACGATTTATTCAAC  GCCCCCCGGATCCCCTGACTCTTATACACAAGT  ATTTTTGGATCCGCGAGGAAGCGATGCCTGC  NNCTTAAGCACACAGGAAACAGCTATGACCATGATTACGG  NNGGATCCCACCTGGAAGATCAGATCCTGGAAAACG  GAATAATCTAGACCTGGTGTCCCTGTTGATACC  GCTTATTCTAGATTATTCAGGCGTAGCACCAGG  CGTGTTGTGAAATGTTGGGTTAAGT  AACCCACTCCCATGGTGTGACGGGC  GTCATTTCGAACCCCAGAGTCC  CGTTGGGATTGCGGATAAATCGGTAAG  TAATACGACTCACTATAGGGAAAAAAAACCAGCAGGTATAATCTGCTG  GATGAAGCAAGGGGGTGC  TAATACGACTCACTATAGGCCTGGCAGTTCCCTACTCTCG  CGGCAGTAGCGCGGTG  TAATACGACTCACTATAGGGCATCGGCAGGGTCATC  AGGTGACCTCTTAAGATGGTAACGTTC  AGGCTGCGCAACTGTTGGG  AGGTGACCTCTTAAGCACACAGG  CTGACTCTTATACACAAGTAGCGTCCTG  NNGAATTCAAGTGCCTTCCCATCAAAAAAATATTCTCAACATAAAAAACTTTGTGTAATACTTGTAACGAGGTGACCTCTTAAGATGGTAACG  CGACTCCGCGGCTGTCG  CAGCTGGTCAACTTTAGCGTTCAGAG | Partially-randomized primer  pDH700-construction  pDH700-construction  pDH658-construction  pDH658-construction  pDH795-construction  pDH795-construction  pDH798/804-construction  pDH798/804-construction  pDH812-construction  pDH812-construction  pDH682-construction  pDH682-construction  pDH883-construction  pDH883-construction  RT-PCR, 16S rRNA  RT-PCR, 16S rRNA  Tn5-specific primer  IS*50* Primer Ext.  SgrS in vitro transcrip. template  Reverse primer for above  5S in vitro transcrip. template  Rev primer for above  T’ase in vitro transcrip. template  Reverse primer for above  Reverse primer, RT-PCR of IS*50*-*lacZ* TCF  Forward primer for above: Readthrough+T1  Forward primer for above: Readthrough only  pDH908-construction  pDH908-construction  lpp Primer Ext. |
